# A dual mechanism of activation of the Sonic Hedgehog pathway in anaplastic thyroid cancer: crosstalk with RAS-BRAF-MEK pathway and ligand secretion by tumor stroma

**DOI:** 10.18632/oncotarget.23388

**Published:** 2017-12-17

**Authors:** Alessia Parascandolo, Mikko O. Laukkanen, Nancy De Rosa, Clara Ugolini, Maria Carmela Cantisani, Anna Maria Cirafici, Fulvio Basolo, Massimo Santoro, Maria Domenica Castellone

**Affiliations:** ^1^ IRCCS SDN, Naples, Italy; ^2^ Department of Molecular Medicine and Medical Biotechnologies, University Federico II, Naples, Italy; ^3^ Department of Laboratory Medicine Section of Pathology Azienda Ospedaliero-Universitaria Pisana, Pisa, Italy; ^4^ Istitute of Experimental Endocrinology and Oncology “G. Salvatore” (IEOS), C.N.R., Naples, Italy; ^5^ Department of Surgical, Medical, Molecular Pathology and Critical Area, University of Pisa, Pisa, Italy

**Keywords:** hedgehog signaling, thyroid cancer, tumor stroma, MSC, co-culture

## Abstract

Sonic Hedgehog (Shh) pathway regulates embryonic development of different organs including the thyroid gland. The aberrant activation of Shh signaling has been found in several types of cancer and according to recent evidences it represents an important regulator of tumor-stroma interaction. In this study, we have analyzed expression, activation and molecular mechanisms regulating the Shh pathway and its involvement in the modulation of tumor stroma interaction in anaplastic thyroid cancer (ATC) cells. Our results suggest that Shh signaling undergoes a dual mechanism of induction in ATC cells: 1) a basal non-canonical Smo-dependent activation of Gli transcription factor that is partly caused by interaction with the RAS/BRAF/MEK oncogenic pathway and is characterized by the absence of Shh ligand expression in thyroid cancer cells and 2) a paracrine response of cancer cells to Shh ligand secreted by tumor stroma (fibroblasts and mesenchymal stromal cells, MSCs) inducing cancer cell migration and *in vitro* tumorigenesis. Our data therefore suggest Shh as a potential novel therapeutic target in aggressive thyroid cancers.

## INTRODUCTION

Thyroid cancer represents 2.6% of all new cancers, when epithelial skin cancers are excluded [1]. Anaplastic thyroid carcinoma (ATC), the most aggressive form of thyroid malignancies, accounts for 1–2% of all thyroid tumors. ATC can arise *de novo* or result in as the last step of the gradual thyroid cancer progression from a well-differentiated tumor. Although multiple genetic events have been identified in ATC, such as *TP53* loss of function mutations, activating point mutations of *RAS*, *BRAF*, and *PI3K*-*AKT*, as well as mutations in the *TERT* promoter inducing TERT over-expression [2, 3], no therapy has been demonstrated successful against this disease that still has a mean survival from diagnosis of about six months.

The Sonic Hedgehog (Shh) pathway plays an important role in regulating cell growth, differentiation and tissue patterning during normal human embryonic development. Shh can act as morphogen in developing tissues in a dose-dependent manner through the induction of different effects on various cell types and it may act as a mitogen that regulates cell proliferation of developing organs [4]. Shh signaling is mediated at the cell membrane by two receptors: a 12-span-transmembrane protein named Patched (Ptch) and a seven-span-transmembrane receptor named Smoothened (Smo) that show dynamic trafficking in primary cilium, a slim microtubule-based organelle that protrudes from the cellular surface, depending on Shh ligand binding [5]. Shh ligands binding to Ptch receptor triggers a cascade of downstream events that culminates in the activation of the zinc finger transcription factors glioma-associated oncogene Gli. Activated Gli proteins (GliA) translocate into the nucleus where they mediate the transcription of different target genes (such as D-type cyclin, Bcl2, VEGF, Myc, snail family zinc finger Snail and Nanog) [6]. In adults, the Shh pathway is mainly quiescent but has important functions in the regulation of tissue homeostasis, continuous renewal and repair of adult tissues and stem cell maintenance [7]. Interestingly, the Shh signaling pathway has been recently recognized as one of the most important signaling pathways involved in human cancer where it promotes tumor cell growth, enables proliferation of tumor stem cells and regulates tumor-stroma interaction [8]. Different mechanisms of Shh pathway activation have been proposed in cancer [6, 9, 10]. A ligand independent Shh pathway activation has been described in some familial cancers, such as basal cell carcinoma, medulloblastoma and rhabdomyosarcoma, as a consequence of genetic aberrations, targeting mainly the Ptch inhibitory receptor [11]. Ligand-independent non-canonical Shh pathway activation has been reported in other tumor models as a result of a crosstalk with different tumorigenic pathways [12]. Finally, a ligand-dependent autocrine or paracrine activation has been involved in tumor progression and maintenance of several cancers, including gastrointestinal, lung, breast and prostate cancers. The paracrine mechanism can either be due to Shh secretion by cancer cells promotion of Gli transcriptional activity in stromal cells (direct paracrine), or to Shh ligand secretion by stroma activating the pathway in cancer cells (reverse paracrine mechanism) [13].

In thyroid, Shh is a regulator of embryonic development by governing symmetric bilobation of the thyroid gland and repressing inappropriate thyroid differentiation in embryonic tissues outside the thyroglossal duct [14]. Moreover, genetic deletion of *Shh* causes hemiagenesis and ectopic development of the thyroid gland in a mouse model [14]. Recent studies have indicated a wide expression of Shh pathway components such as Ptch and Smo receptors and the transcription factor Gli1 in PTC papillary thyroid carcinoma (PTC), ATC and in benign follicular thyroid adenoma (FTA) suggesting that the Shh pathway activation occurs at an early phase of thyroid tumor development. The expression of Shh pathway components has also been found in ATC suggesting that an aberrant activation of this pathway can be involved in anaplastic thyroid transformation [15]. Finally, an increased expression of the Shh signaling component in medullary thyroid cancer (MTC) with respect to normal thyroid tissue has been demonstrated [16]. Nonetheless, the activation of the Shh pathway does not seem to be causative of thyroid neoplastic transformation, but it rather seems to promote thyroid tumorigenesis and progression in collaboration with other oncogenic pathways [17] although the mechanisms of this activation have not been described yet.

Here we have studied the role of Shh pathway in ATC and its involvement in tumor interaction with microenvironment. We have analyzed the expression and activation of Shh signaling components in ATC cells and tumor samples, studied the molecular mechanisms regulating Shh pathway activation and investigated the role of Shh signaling in regulating tumor stroma interaction.

## RESULTS

### Thyroid tumors and cell lines express Shh pathway molecules but not Shh ligand

**Figure 1:**
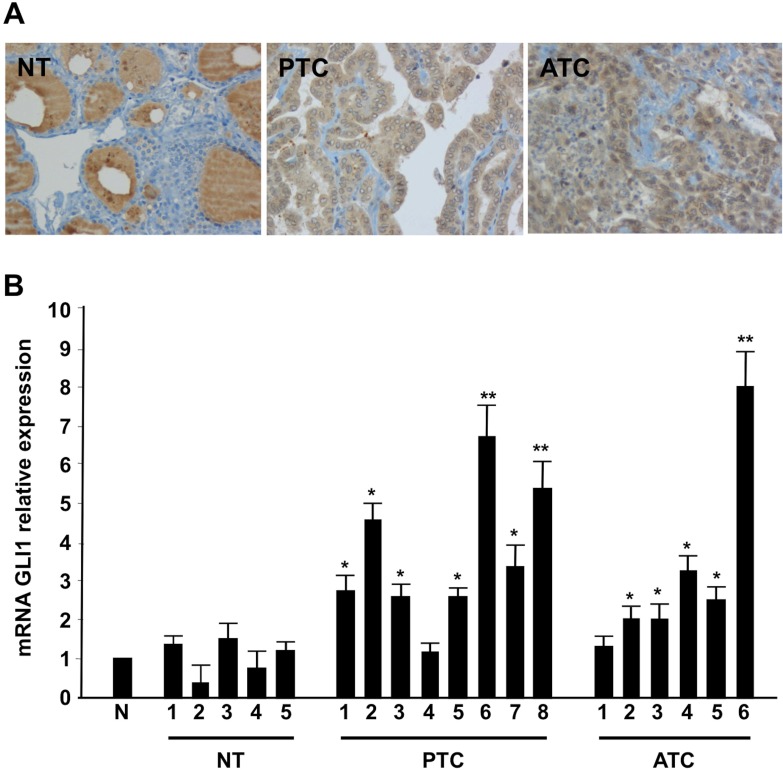
Shh signaling components are up-regulated in thyroid tumors (**A)** Representative histological sections from normal thyroid (NT), PTC and ATC incubated with a mouse monoclonal anti-Gli1 antibody. ATC samples showed intense immunoreactivity for Gli1, PTC samples showed a mild expression of Gli1 while normal thyroid tissues were completely negative. Negative controls were performed in all cases using isotype-matching control antibodies. (**B)** Q-RT-PCR of Gli1 was performed in five normal thyroids (NT), eight PTCs and six ATCs tumor samples. The fold induction was calculated using the pool of the five normal thyroid tissues as control (N). The results are the average of three independent experiments. Error bars represent standard deviations of three independent experiments. ^**^p = ≤ 0.01; ^*^p = ≤ 0.05.

Shh pathway signaling culminates into the activation of the glioma-associated zinc finger transcription factor Gli [6]. Here we evaluated by immunohistochemistry Gli1 expression levels in a panel of 66 human tissues including: five normal thyroids, 11 benign adenomas, 10 papillary thyroid cancers (PTCs), 10 medullary thyroid cancers (MTCs), 14 follicular thyroid cancers (FTCs), and 16 anaplastic thyroid cancers (ATCs). Normal thyroids were all negative for Gli1, whereas tumors were positive, with a higher proportion of positive cells in ATC than in adenoma and FTC (Figure 1A and Table 1). Interestingly, among PTCs, the positivity in tall cell variant samples (PTC-TCV) was particularly high, whereas it was lower in the follicular variant (PTC-FV) and did not correlate with tumor size or age of the patients (data not shown). The Gli1 up-regulation also occurred at the RNA level, as examined by Q-RT-PCR in a set of normal, PTC and ATC tissues (Figure 1B).

**Table 1:** Thyroid samples of different histotype were stained for Gli1 expression

Histotype	Number of cases	Positive cases	% positive cells (mean)
PTC	10	9	45
ATC	16	16	69
MTC	10	10	66
FTC	14	10	46
Adenoma	11	8	42
Normal	5	0	

The majority of tumor samples resulted positive for Gli1 while all normal cases were negative. Average positive cells were calculated for each histotype.

We then performed Q-RT-PCR and immunoblotting experiments of Shh pathway components in a panel of papillary (TPC1, BCPAP) and anaplastic (SW1736, 8505C, CAL62, OCUT1) thyroid cancer cells compared to immortalized NTHY cell line (derived from non-tumoral tissue). Our data indicate that all the thyroid cancer cells were positive for Gli1, Smo and Ptch expression, the latter being downregulated only in BCPAP cells (Supplementary Figure 1A–1C). Interestingly, we were unable to detect any Shh expression in thyroid cells, tested by both Western blotting (Supplementary Figure 1D), as well as Q-RT-PCR (data not shown). Thereby, our expression data suggest lack of Shh ligand expression in thyroid cancer cells but increased expression of Shh pathway molecules.

### Shh pathway is active in thyroid cancer cells

**Figure 2:**
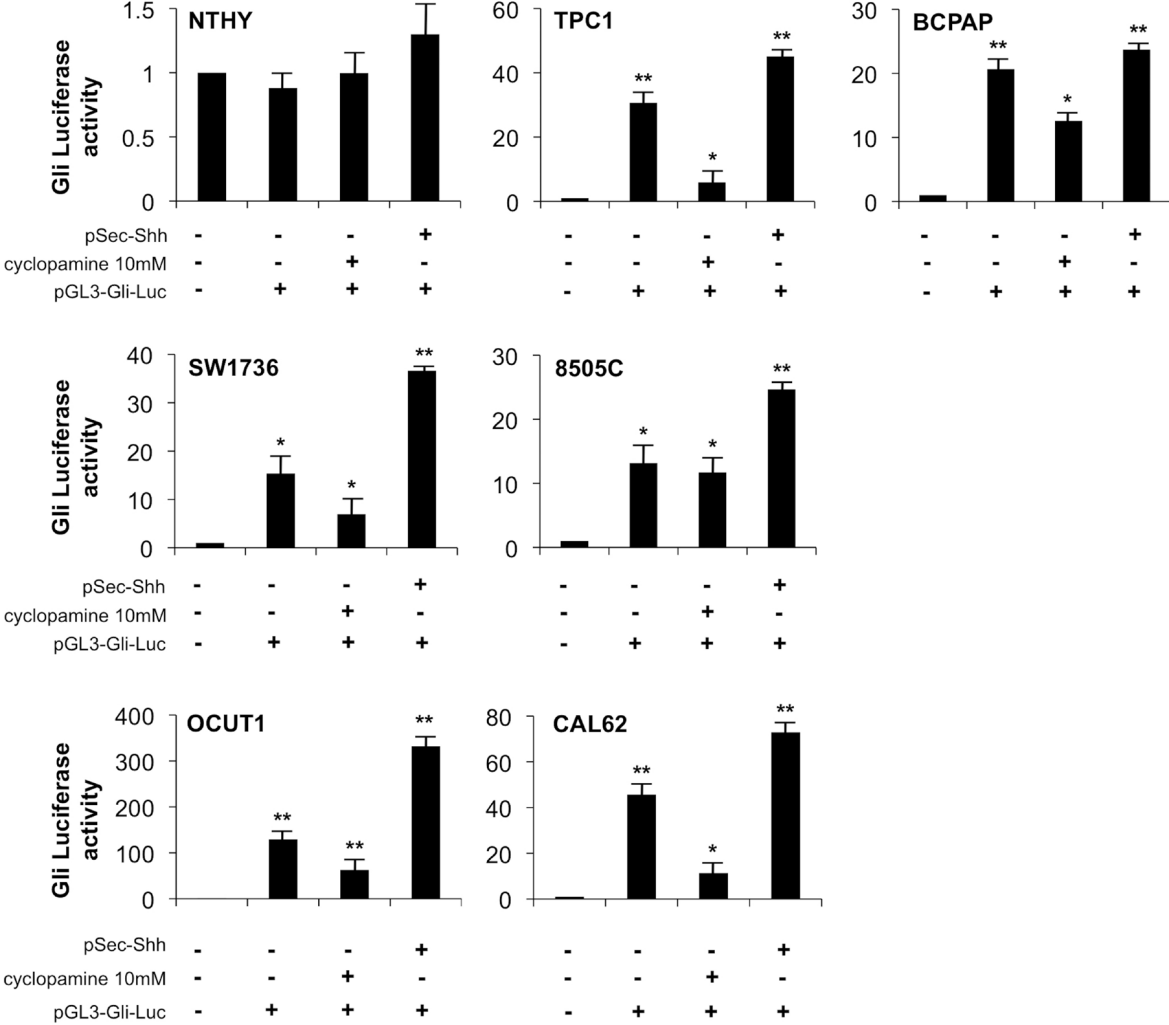
Shh pathway is active in thyroid cancer cells and it is responsive to cyclopamine Thyroid cells were transfected with Gli-Luc reporter (pGL3-Gli-Luc) and 48 hours later Gli transcription factors activation was measured by Luciferase assay. Transfected cells were also treated with cyclopamine 10 mM for 24 h and stimulated with Shh ligand (pSec-Shh produced by HEK293T cells). Error bars represent standard deviations of experimental triplicates. ^**^p = ≤ 0.01.

To test if the increased Shh pathway molecules corresponded to increased functionality of the Shh signaling pathway in our model, we transfected the thyroid cell panel with a Gli-Luc reporter plasmid (characterized by the luciferase cDNA under the control of eight tandem Gli1-responsive elements, Supplementary Figure 2A lower panel). Moreover, to study the mechanism of activation of the Shh signaling in thyroid cancer cells and to verify whether the signaling is mediated by the membrane receptor Smo, we took advantage of using the drug cyclopamine, a commercially available Smo inhibitor. Thyroid cells were transfected with Gli-luc, stimulated with recombinant Shh produced by HEK-293T cells (pSec-Shh) and incubated with or without cyclopamine. Interestingly, while normal NTHY cells showed lack of Gli reporter activity even in the presence of Shh ligand stimulation, PTC (TPC1 and BCPAP) and ATC (SW1736, 8505c, OCUT1, and CAL2) cell lines showed robust constitutive Gli reporter activation that was further increased by Shh stimulation and inhibited by cyclopamine (Figure 2). Our results hence demonstrate a cyclopamine-responsive basal activation of Shh pathway in thyroid cancer cells that is further inducible by stimulation with Shh ligand.

**Figure 3:**
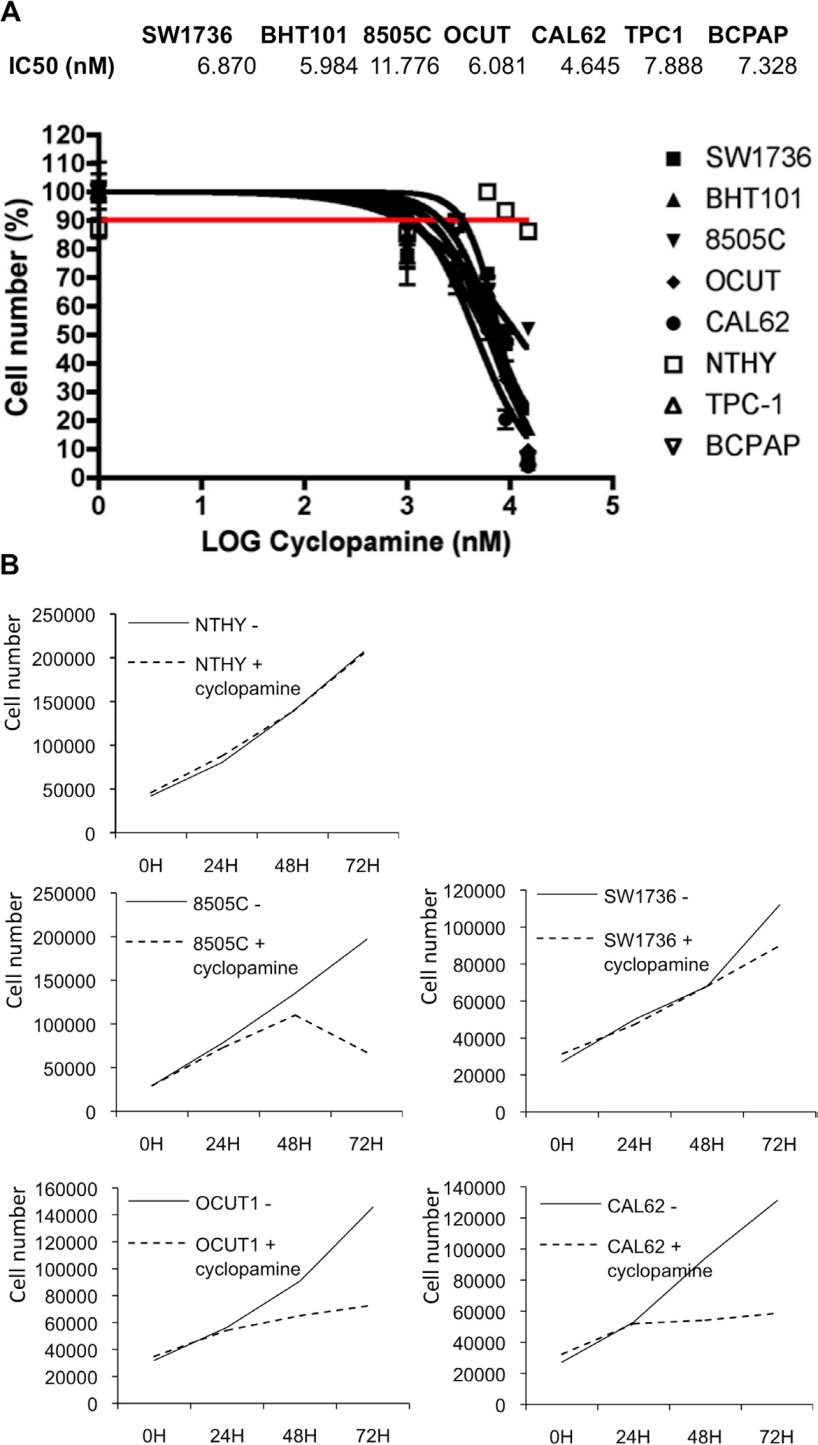
Thyroid cancer cells growth is sensitive to cyclopamine (**A**) IC50 (inhibitory concentration 50) of cyclopamine was assayed in thyroid cells treated with different doses of the drug (1–5–10–20 mM) for 72 hours. Cells were counted in triplicate. (**B**) For growth curve, 3 × 10^4^ thyroid cells were plated and treated with cyclopamine 10 mM. Cells were counted in triplicate every 24 hours. At 72 hours the number of cells was reduced compared to untreated cells. Three independent determinations were performed in triplicate.

To dissect the dose response of cyclopamine on thyroid cells we measured cyclopamine-inhibitory concentration 50 (IC50) for proliferation of thyroid cancer cell lines and NTHY control cells by using increasing doses of the compound (Figure 3A). Treatment of cancer cells with cyclopamine reduced proliferation with an IC50 ranging between 4.64 μM and 11.77 mM, whereas 20 mM of cyclopamine did not reach IC50 in NTHY cells thereby suggesting specificity of the drug for transformed cells.

Based on our IC50 measurements we performed a growth curve by treating thyroid cells with a 10 mM concentration of cyclopamine for 72 h. As shown in Figure 3B, drug treatment decreased the growth of the cancer cells of approximately 50% (8505C, OCUT1, CAL62) and approximately 20% (SW1736) as compared to untreated cells, whereas the same treatment did not have any effect on NTHY cells proliferation.

### RAS-BRAF pathway crosstalk increases Gli activity

**Figure 4:**
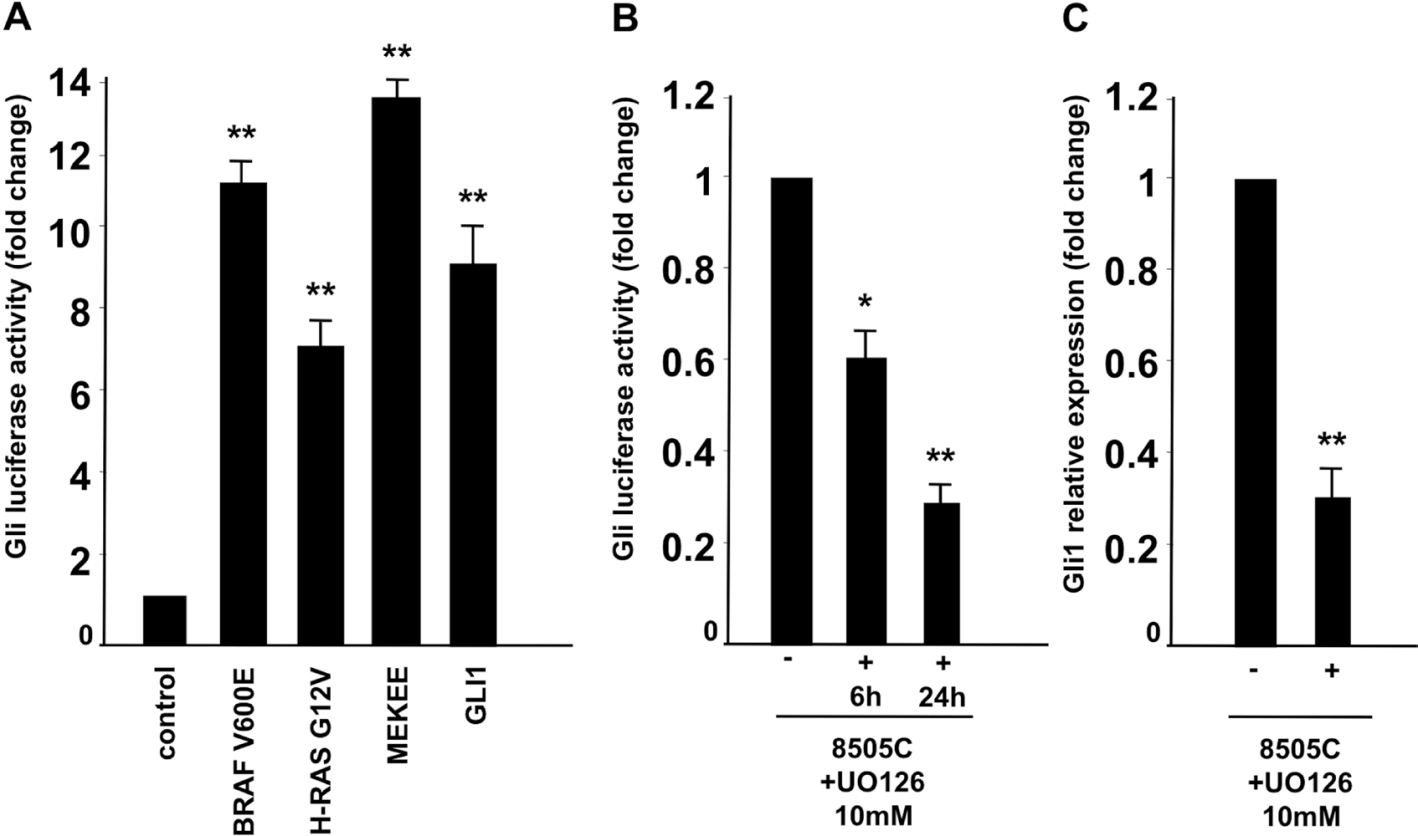
Oncogenic RAS/BRAF/MEK pathway activates Shh signaling (**A**) HEK293T cells were transfected with Gli-Luc reporter (pGL3-Gli-Luc) together with BRAF V600E, HRAS G12V and MEK S218E/S222E active forms and luciferase activity was measured 48 hours after transfection. Gli1 transfection was used as positive control. (**B**, **C**). Inhibition of MEK impaired Gli luc activation. 8505C cells were transfected with Gli-Luc reporter (pGL3-Gli-Luc). After 24 hours cells were treated with U0126 at 10 μM for 6 and 24 hours and Shh activation was evaluated by Luciferase assay (B) and Q-RT-PCR to test Gli1 relative expression (C). Error bars represent standard deviations of experimental triplicates. ^**^p = ≤ 0.01; ^*^p = ≤ 0.05.

Because our expression studies showed undetectable Shh ligand production in thyroid cancer cells, we hypothesized that the Smo receptor could be activated in a non-canonical ligand-independent manner. Several reports have described a crosstalk between Shh signaling and other signal transduction pathways in different types of cancer [12]. Based on previous reports ATC cancer cells are positive for BRAF V600E (8505C, SW1746, OCUT1) or KRAS G12R (CAL62) oncogenic mutation [18]. Therefore, we tested whether active forms of RAS, BRAF and MEK1 could stimulate the Shh pathway by performing a Gli-Luc assay in embryonic kidney HEK293T cells upon oncogenic transfection. Our results, shown in Figure 4A suggest that BRAF V600E, HRAS GV12 and MEK-S218E/S222E (MEKEE) mutants are able to boost Gli Luc activity at a similar level of Gli1 used as positive control. The data was confirmed by performing a Gli-Luc assay in a rat-derived differentiated thyroid follicular cell line (PC-Cl3) stably expressing BRAF (PC-BRAF V600E) or HRAS (HRAS G12V) oncogenes compared to wild type PC-Cl3 cells (Supplementary Figure 2A). The Gli-luc activation upon RAS/BRAF expression is most likely the result of increased Gli transcription, as PC-BRAF and PC-HRAS cells show increased expression of Gli1 mRNA with respect to parental un-transfected cells, tested by Q-RT-PCR in Supplementary Figure 2B.

Our data suggest therefore an interaction between Shh and BRAF/RAS/MEK in ATC cell lines. To confirm this hypothesis we took advantage of specific MEK1/2 inhibitor (U0126) that was used at a 10 μM concentration on thyroid cancer cells. As shown in Figure 4B, Gli-Luc was strongly reduced in 8505C cells upon U0126 treatment, in a time dependent manner (6 and 24 hours). Similarly, *Gli1* RNA expression levels were reduced in 8505C cells treated with U0126, as shown in Figure 4C suggesting that RAS pathway increases Gli activity by boosting Gli1 mRNA production. All these findings hence suggest that oncogenic RAS/BRAF/MEK pathway influences Shh pathway activation in thyroid cancer cells generating a ligand independent (cyclopamine-sensitive), non-canonical mechanism of activation.

### Thyroid cancer stroma as a source of Shh ligand

**Figure 5:**
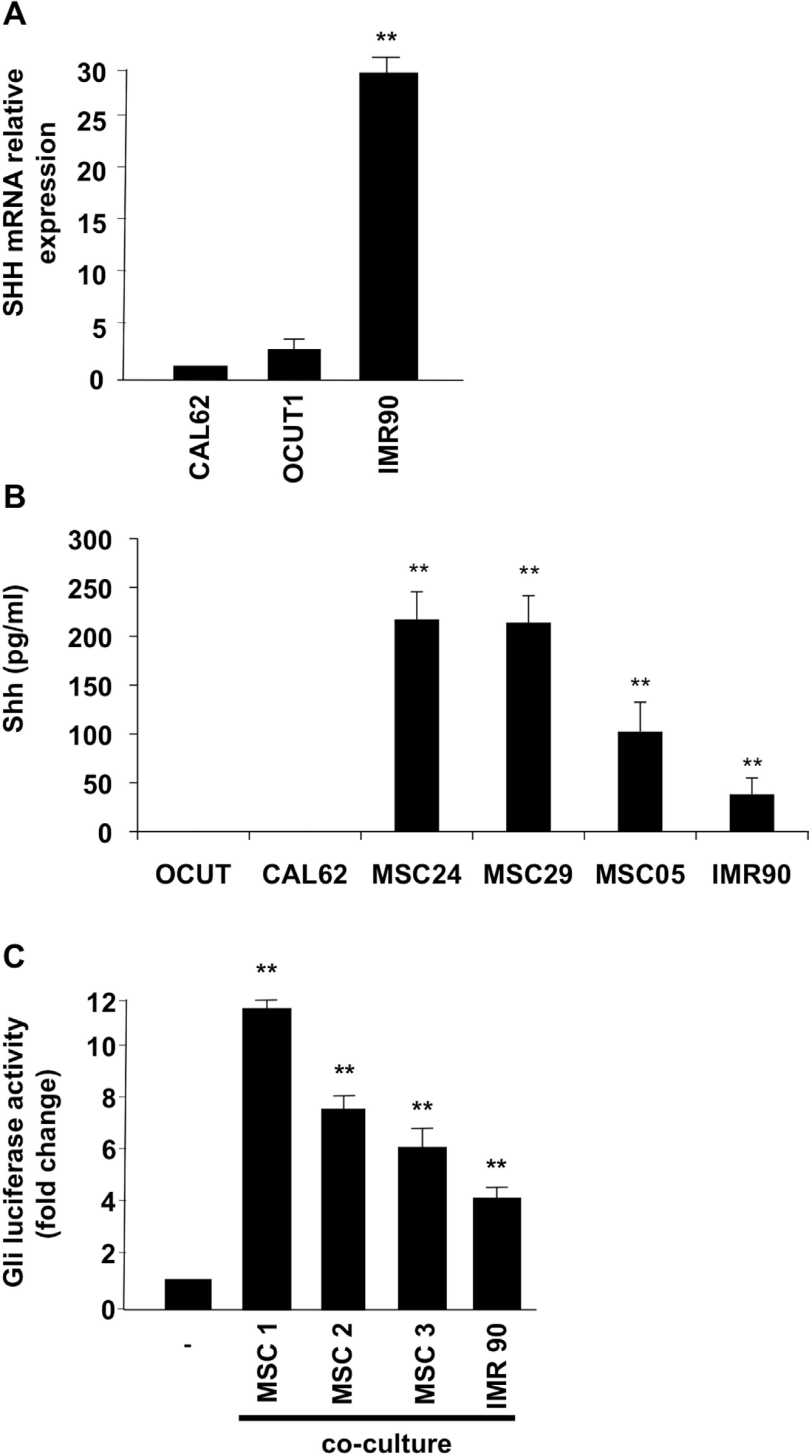
Stromal cells produce Shh ligand and induce signaling activation in ATC cells (**A**) Shh mRNA expression levels were evaluated by Q-RT-PCR in CAL62, OCUT1 and IMR90 cells. (**B)** Elisa assay was performed on the supernatant of thyroid and stromal cells to measure the Shh production. Error bars represent standard deviations of experimental triplicates. (**C**) 3 × 10^4^ Cal62 cells were plated on cover glass and transfected with Gli-Luc reporter (pGL3-Gli-Luc). After 24 hours cells cover glasses were put in co-culture with mesenchymal cells (MSC) or IMR90 fibroblasts for 24 hours. Shh pathway activation was evaluated by Luciferase assay. Error bars represent standard deviations of different experiments. ^**^p = ≤ 0.01; ^*^p = ≤ 0.05.

Because our data (shown in Figure 2) suggested that, although there is a basal ligand independent Shh signaling activation in thyroid cells, the signaling is still responsive to exogenous ligand, we investigated whether the thyroid stroma could be a source of Shh ligand production. It has been described in fact that, in several cancer models, Shh signaling is a crucial mediator in tumor-stroma interaction [19, 20]. To this purpose we used IMR90 fibroblasts, as well as three different human primary cultures of thyroid mesenchymal stromal cells (MSC) [21]. Interestingly, our data demonstrate robust *Shh* mRNA expression levels in IMR90 fibroblasts (compared to negative OCUT1 and CAL62 cancer cells) (Figure 5A) and high-level secretion of Shh ligand (measured through an ELISA assay) in IMR90 and thyroid MSCs as compared to non-detectable values in OCUT1 and CAL62 cells (Figure 5B). To test whether the ligand produced by stromal cells could activate Shh pathway in cancer cells, we co-cultured OCUT1 and CAL62 cells with IMR90 and MSC, and performed a Gli-Luc assay. Our data shown in Figure 5C indicate that stroma co-culture increases Gli transcriptional activity in CAL62 cells suggesting a paracrine Shh signaling activation. These data were paralleled by stroma-stimulated increased Gli1 and Gli2 protein expression in the nuclear compartment and by increased Smo receptor protein levels in CAL62 and OCUT1 cancer cells with respect to non-stimulated control cells (Supplementary Figure 3A–3C).

### Thyroid cancer stroma secreted Shh mediates tumor-stroma interaction in thyroid cancer cells and supports cancer cells invasion, migration, and growth in non-adherent conditions

**Figure 6:**
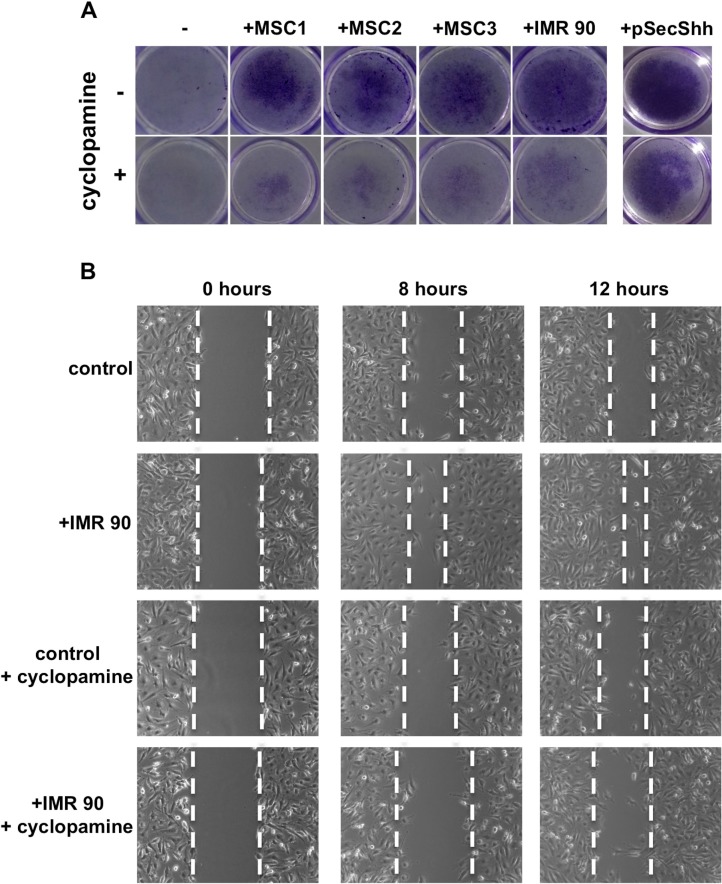
Shh pathway controls the ability of stromal cells to stimulate matrigel invasion and increase migration ability of ATC cells (**A**) 2 × 10^5^ Stromal cells were plated on the bottom of wells while OCUT1 or CAL62 cells (2 × 10^5^) were plated into the upper chamber. The lower chamber was filled with complete medium. Cyclopamine (10 mM) was added to cancer cells (upper chamber) and to stromal cells (bottom of well). After 48 hours cells were fixed, stained and photographed. Cancer cells, in the presence of stromal cells, showed increased invasion ability compared to control cells. Cyclopamine treatment impaired the effect of stromal cells on cancer cells invasion abilities. Shh stimulation was used as a positive control. (**B**) Cells were plated and a scratch wound was inflicted on cellular monolayer. Cancer cells were stimulated with conditioned medium derived from IMR90 and photographed at different time points. Stroma stimulated cancer cells showed increased migration ability compared to control un-stimulated cells and were affected by cyclopamine treatment (10 mM).

We then studied the functional effect of stromal fibroblasts secreted Shh on thyroid cancer cells. Interestingly, cell proliferation analysis in the presence of fibroblast conditioned medium did not show significant differences between stroma stimulated cancer cells and untreated control cells suggesting that the paracrine Shh activation does not have a role on thyroid cancer cells proliferation (data not shown). We therefore investigated the ability of OCUT1 and CAL62 cancer cells to migrate and invade in the presence of IMR90 and MSC cells. To this aim we performed a Matrigel invasion assay by plating IMR90 and MSCs stromal cells into the bottom of a transwell dish allowing OCUT1 and CAL62 cells seeded into the top chamber to migrate towards the stroma. As shown in Figure 6A (upper panels) the presence of stromal cells stimulated cancer cell invasion (the stimulation with secreted Shh ligand, pSecShh, was used as positive control). Importantly, the inhibition of Smo with 10 mM cyclopamine (Figure 6A, lower panels) reduced the ability of stromal cells to attract cancer cells to invade, indicating a role for the paracrine cancer-stroma Shh pathway activation in regulating invasive properties of thyroid cancer cells.

To confirm the stimulatory role of paracrine Shh production on cancer cell movement, we performed a wound healing assay by measuring the ability of CAL62 and OCUT1 cells to close an artificial wound in the presence of stromal cell conditioned medium. Our results, shown in Figure 6B, indicate that cancer cells stimulated with stroma conditioned medium have increased cell migration ability with respect to control non-stimulated cells. Cyclopamine treatment (10 mM) impaired the effect. The migration of control (un-stimulated) cells was similar with and without cyclopamine administration suggesting a stroma-dependent activation of Smo signaling mediated migration (Figure 6B).

**Figure 7:**
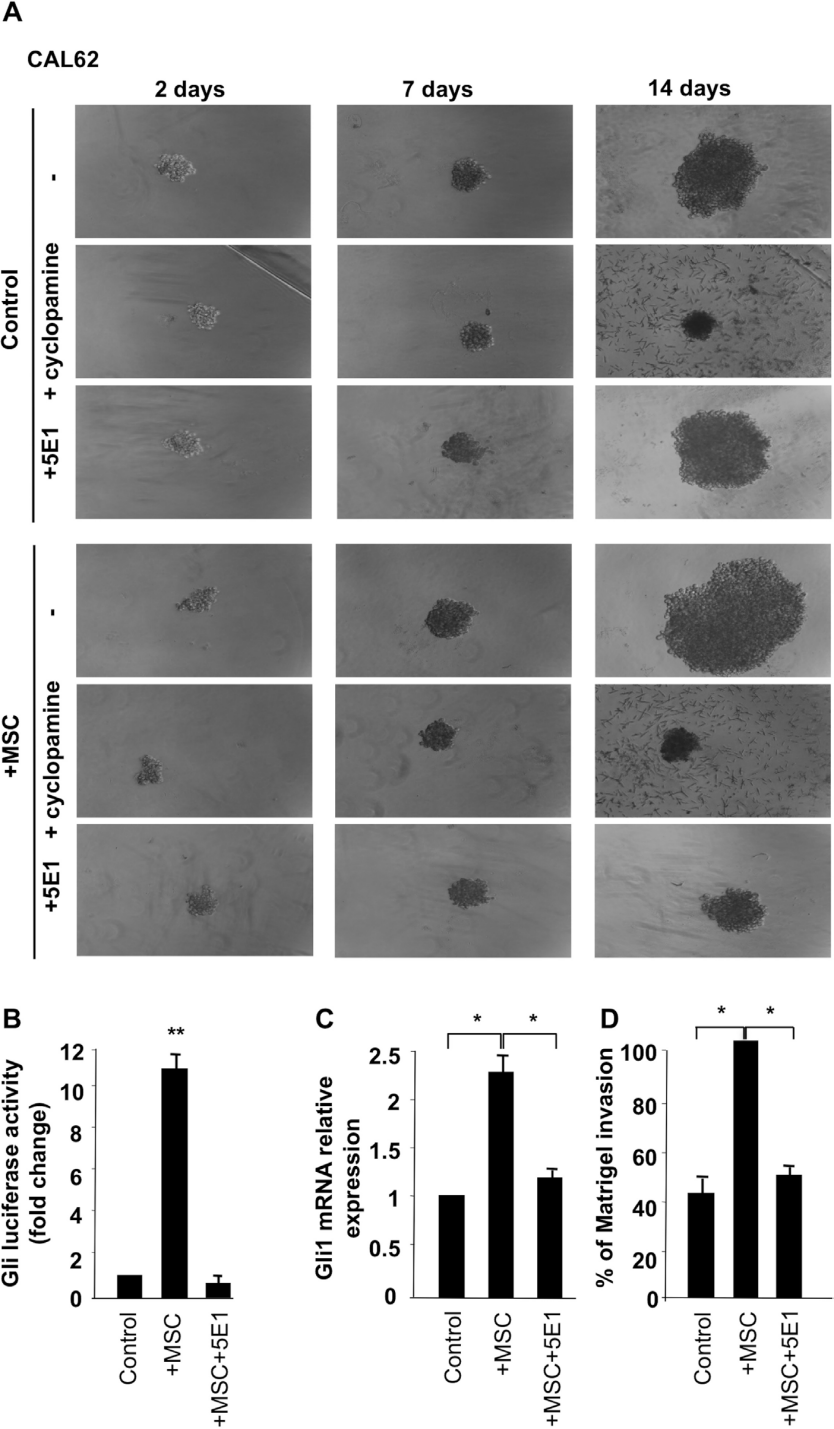
Stromal cells, through production of Shh, support cancer cells growth in non adherent conditions (**A**) CAL62 cells (50 cells/well) were grown on ultra-low-attachment 96-well in low serum medium alone or together with MSC or IMR90 cells (20 cells/wells). Spheroids were photographed after 7 and 14 days. Cancer cells, supported by stromal cells, formed larger spheroids and this ability was impaired by cyclopamine (10 mM) treatment as well as by 5E1 blocking antibody (2.5 μg/ml). Dependence of stromal cells effect on Shh production was tested by performing Gli luciferase assay (**B**), Q-RT-PCR (**C**) and Matrigel assay (**D**) on un-stimulated and MSC stimulated CAL62 cells with and without 5E1 blocking antibody. Error bars represent standard deviations of experimental triplicates. ^**^p = ≤ 0.01; ^*^p = ≤ 0.0.

To model 3-dimensional organization of tumor-stroma interaction, we cultured CAL62 cells alone or together with stromal cells (IMR90 and MSC) under non-adherent and low serum conditions. As shown in Figure 7A, CAL62 cancer cells were able to form spheroid-like structures, whereas IMR90 and MSCs alone did not (data not shown). Of note, co-culture of thyroid cancer cells with MSCs resulted in larger spheroid formation that was almost completely inhibited by 10 mM cyclopamine treatment (Figure 7A). Intriguingly, the use of a Shh blocking antibody (5E1) in the co-culture, dramatically reduced spheroids formations although it did not have any effect on cancer cells alone (Figure 7A), strengthening the evidence that stroma cells by paracrine Shh production support thyroid cancer cell ability to grow in non- adherent/low serum conditions. The dependence of CAL62 cancer cells (CAL62) on Shh production from stroma was confirmed by performing Gli-Luc, Gli expression and Matrigel assay on MSC stimulated cells in the presence of 5E1 (Shh blocking) antibody (Figure 7B–7D).

**Figure 8:**
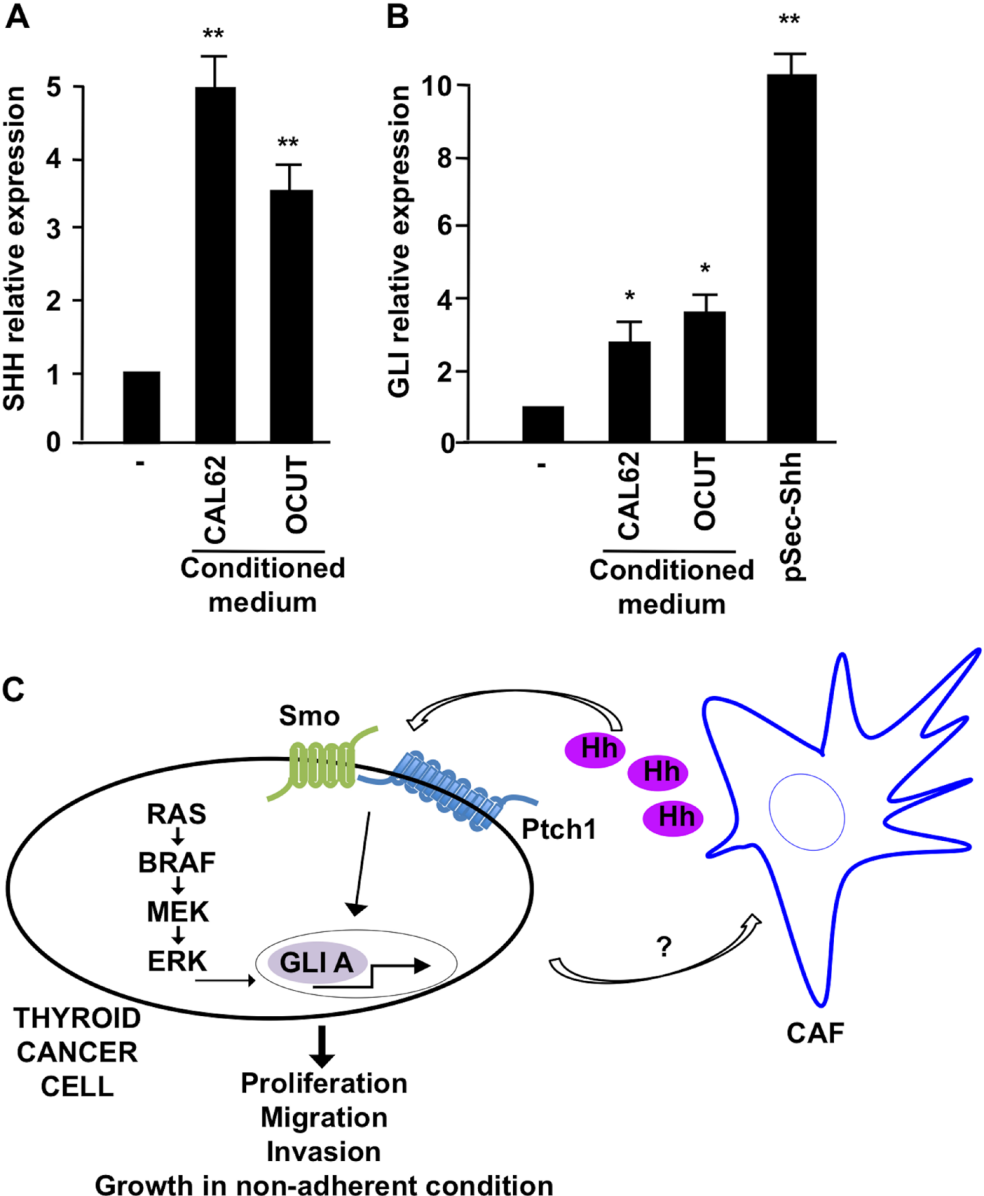
Effect of CAL62 and OCUT cells on IMR90 fibroblasts (**A**) Q-RT-PCR of Shh in IMR90 upon stimulation with conditioned medium derived from CAL62 and OCUT cells. (**B**) Q-RT-PCR measuring the expression of Gli1 in IMR90 stimulated with cancer cells (CAL62/OCUT) conditioned medium. Shh ligand stimulation was used as positive control. ^**^p = ≤ 0.01; ^*^p = ≤ 0.05. (**C**) Schematic representation of Shh pathway activation mechanisms in thyroid cancer cells.

To finally test whether there would be a bi-directional Shh signaling between cancer and stroma cells, we stimulated IMR90 fibroblasts with the supernatant derived from OCUT1 and CAL62 cells and tested if cancer cells could influence their stromal counterpart, by affecting the amount of Shh production. Compellingly, in the presence of cancer cell derived conditioned medium, IMR90 fibroblasts showed an increased level of Shh mRNA expression (Figure 8A) and *Gli* mRNA transcription, target of Shh signaling activation (Figure 8B), suggesting that paracrine factors produced by cancer cells can increase Shh production levels in stroma, which then potentiates tumor expansion, local migration, and invasion.

## DISCUSSION

Aberrant activation of Shh pathway leads to malignancy. Previous studies have suggested that components of the Shh pathway are expressed at high levels in tumors, particularly in aggressive and undifferentiated cancer types [6]. Here we have investigated the activity of this pathway in anaplastic thyroid cancer that represents the histotype of thyroid cancer with the worst prognosis. A growing body of evidences indicates the stroma as a key player in tumorigenesis to support the growth, maintenance and progression of cancer. In thyroid cancers, stroma composition changes among the different histotypes being mainly characterized by immune/inflammatory cells in PTC and by a desmoplastic stromal reaction in ATC and MTC as well as in metastatic PTC, where it correlates to aggressiveness and lymph node metastasis [22–26]. Recent reports in cancer models have suggested a crucial role for the Shh pathway in tumor-stroma interaction. The paracrine Shh ligand production from cancer cells has been described to stimulate peritumoral stroma to produce growth and survival signals such as insulin-like growth factor (IGF), Wnt and VEGF, that enhance cancer progression [13, 19, 27, 28]. Similarly, a variant of this Shh signaling is the “reverse paracrine signaling” where the Shh ligand is secreted from the stroma and is received by the tumor cells. However, this mechanism has been described until now only in hematological malignancies, such as leukemia, multiple myeloma, and B-cell lymphoma [6, 7].

The importance of Shh pathway in proper thyroid development has been deeply studied [14], but only recently expression studies have demonstrated the presence of components of the Shh pathway in thyroid tumors of different histotypes, suggesting an activation of Shh signaling at early phases of thyroid tumorigenesis [15, 16, 29, 30]. Moreover, a recent study demonstrated that the activation of the Shh pathway is not the cause of thyroid neoplastic transformation but it rather promotes thyroid tumorigenesis and progression in collaboration with other oncogenic pathways [17]. However, the mechanism of Shh action in thyroid tumours is still largely uncharacterized.

Here we have addressed the role and mechanism of activation of the Sonic Hedgehog pathway in ATC and its involvement in tumor-stroma interaction. Importantly, our experiments demonstrate absence of Shh ligand in thyroid cancer cells with simultaneous constitutive activation of the pathway as shown by Gli transcriptional activity and sensitivity to cyclopamine treatment, suggesting that in thyroid cancer cells the Shh pathway is activated in a ligand-independent but Smo-dependent manner. To this aim, we tested the possibility of a cross-talk between the Shh pathway and the RAS/BRAF oncogenic signaling cascade active in most thyroid cancer cells. Our data suggest indeed that transfection of RAS/BRAF/MEK oncogenes is able to directly activate Gli expression and transcription while MEK specific inhibition (with U0126) reduced the activity of the pathway in BRAF mutated cells. In addition, because our data indicated that the Shh pathway could be stimulated by exogenous Shh administration to thyroid cancer cells, we hypothesized a ligand production from stromal cells potentiating pathway activation in cancer cells (reverse paracrine). Our co-culture experiments and stimulation with conditioned-medium suggest indeed that stromal cells (IMR90 and MSCs) support thyroid cancer cells invasion, migration and non-adherent growth abilities. These effects are Smo dependent, because impaired by cyclopamine and Shh dependent because blocked by 5E1 antibody. Interestingly, we also observed an increased Shh production in fibroblasts stimulated with conditioned medium derived from cancer cells, suggesting a bi-directional paracrine effect (Figure 8C).

Overall, in this study, we show a dual mechanism of activation of the Shh pathway in thyroid tumors: a ligand-independent activation, caused by interaction with RAS/BRAF/MEK oncogenic cascade, and a ligand-dependent activation sustained by ligand (Shh) secretion from fibrotic stroma, promoting cancer cell migration and invasion. While the first mechanism is common to PTC and ATC (that share driver lesions in components of this cascade: eg. RAS and BRAF), the second one might be more typical of ATC based on the increased response of the ATC cell lines to ligand triggering. Accordingly, several GLI1 target genes (including TGFβ, TWIST, CDC25B, ZEB) have been described in previous reports as part of the ATC gene expression signature [3], therefore suggesting that targeting the Shh signaling will lead to inhibition of a number of genes known to induce cell proliferation, migration, invasion and metastatization in ATC.

A combined inhibition therapy of Shh pathway and MEK or AKT, has been shown to produce synergistic effects in reducing melanoma and cholangiocarcinoma cell proliferation *in vitro* [31]. Moreover, Smo inhibitors, in combinatorial strategies with EGFR inhibitors, have been described in several preclinical models. In pancreatic cancer cells for instance, EGFR inhibitor gefinitib in combination with cyclopamine, has shown a tumor growth decrease and apoptotic rate increase [32], whereas in prostate cancer cells, cyclopamine in combination with gefitinib and docetaxel treatment has demonstrated an inhibitory effect on proliferation and invasiveness [33]. Finally, combination treatments targeting both the stroma and the cancer cells with Shh inhibitor IPI-926, together with chemotherapy drug gemcitabine, has shown depletion of stroma and increased drug delivery in a preclinical mouse model of pancreatic cancer proving efficacy of combinatorial strategy against both cancer cells and stroma [9, 34]. In conclusion, our data support the correlation between stroma inhibition and reduced aggressiveness thereby suggesting the possibility to target Shh pathway in thyroid cancer cells as well as in tumor microenvironment in ATC.

## MATERIALS AND METHODS

### Cell lines

TPC1 cells were originally obtained by M. Nagao (Carcinogenesis Division, National Cancer Center Research Institute, Tokyo, Japan). SW1736 were obtained by N.E. Heldin (University Hospital, S-751 85 Uppsala, Swden). BCPAP were obtained by the primary source (N. Fabien, CNRS URA 1454, University of Medecine Lyon-Sud, Oullins, France). 8505C, CAL62 anaplastic carcinoma cells were purchased from DSMZ (Deutsche Sammlung von Mikroorganismen und Zellkulturen GmbH, Braunschweig, Germany); OCUT1 cells were provided by N. Onoda (Osaka University of Medicine, Osaka, Japan) in 2005; NTHY are normal human thyrocytes immortalized by the Large T of SV40 and were obtained from the European Tissue Culture collection [35]. HEK293 cells were from American Type Culture Collection (ATCC). All human epithelial cells grown in either Dulbecco's Modified Eagle Medium (DMEM) or RPMI 1640 medium supplemented with 10% fetal bovine serum. IMR-90 and thyroid MSCs were grown in αMEM supplemented with 10% defined FBS (Hyclone, Logan, UT, USA) and non-essential amino acids (Euroclone, Milano, Italy). All media were supplemented with 2 mM L- glutamine and 100 units/ml penicillin-streptomycin (GIBCO, Waltham, MA, USA). The Fischer rat-derived differentiated thyroid follicular cell line PC Cl 3 was grown in Coon's modified Ham F12 medium supplemented with 5% calf serum and a mixture of six hormones (6H): thyrotropin (10 mU/ml), hydrocortisone (10 nM), insulin (10 μg/ml), apo-transferrin (5 μg/ml), somatostatin (10 ng/ml), and glycyl-histidyl-lysine (10 ng/ml) (Sigma-Aldrich). PC adoptively expressing several oncogenes [36] have been cultured in the same medium as PC but without the 6H.

### Tissue samples

A set of PTC, ATC and normal thyroid tissue samples snap-frozen in liquid nitrogen and maintained at –80°C has been made available by Prof. F. Basolo (University of Pisa, Italy). For all of them, formalin-fixed paraffin-embedded tissue slides were reviewed by 2 pathologists (F. Basolo, C. Ugolini) to ensure diagnosis. Informed consent was obtained from the patients and the study was approved by the institutional review board committee. Tumor size, extra-thyroid invasion, node metastasis, associated thyroid lesions and metastatic deposits were recorded. After surgical resection, tissues were fixed in 10% neutral buffered formalin and embedded in paraffin blocks. Sections (4 microns thick) were stained with hematoxylin and eosin for histological examination.

### Immunohistochemistry

Formalin-fixed and paraffin-embedded 4- to 5-microns-thick tumor sections were deparaffinized, placed in a solution of absolute methanol and 0.3% hydrogen peroxide fore 30 min and treated with blocking serum for 20 min. The slides were incubated overnight with anti-Gli1 monoclonal antibody (Vectostain ABC kits, Vector Laboratories, Inc., Burlingame, CA, USA). As a negative control, tissue slides were incubated with isotype-matched IgG1 control antibodies. The percentage of positive cells for Gli1 staining was evaluated.

### Compounds

For *in vitro* experiments, cyclopamine hydrate was purchased by Sigma-Aldrich (St. Louis, MO, USA). Cyclopamine was dissolved in dimethyl sulfoxide (DMSO) at concentration of 3 mM and stored at –80°C. U0126 was provided by Cell Signaling, (Danvers, MA, USA) and used at a 10 μM concentration. 5E1 blocking antibody was purchased from The Developmental Studies Hybridoma Bank, University of Iowa, USA, and used at the concentration of 2.5 mg/ml.

### Immunoblotting

Protein lysates were prepared according to standard procedures [37]. Briefly, cells were harvested in lysis buffer (50 mM Hepes, pH 7.5, 150 mM NaCl, 10% glycerol, 1% Triton X-100, 1 mM EGTA, 1.5 mM MgCl2, 10 mM NaF, 10 mM sodium pyrophosphate, 1 mM Na3VO4, 10 μg of aprotinin/ml, 10 μg of leupeptin/ml) and clarified by centrifugation at 10,000 × *g*. Nuclear proteins extraction was performed using NE-PERÒ Nuclear and Cytoplasmic Extraction Reagents kit (#78835) (Thermo Scientific, Rockford, IL, USA) according to manufacturer's instructions. Protein concentration was estimated with a modified Bradford assay (Bio-Rad) and lysates were subjected to SDS PAGE. Membranes were probed with the indicated antibodies. Immune complexes were revealed by an enhanced chemiluminescence detection kit (ECL, Amersham Pharmacia Biotech). Signal intensity was quantified with the Phosphorimager (Typhoon 8600, Amersham Pharmacia Biotech) interfaced with the ImageQuant software.

### Antibodies

Anti Shh (#2207) and anti Gli1 (#2553) are rabbit monoclonal antibodies from Cell Signaling Technology. Anti Smo (E-5) sc-166685 (#C1315) mouse monoclonal antibody and anti Gli2 (H-300) sc-28674 (#K2013) rabbit polyclonal antibody are from Santa Cruz Biotechnology (Santa Cruz, CA, USA). Polyclonal antibody anti-Parp (#9542) is from Cell Signaling Technology. Monoclonal anti-tubulin (#T9026) is from Sigma-Aldrich (St Louis, MO, USA). Secondary antibodies coupled to horseradish peroxidase are from Amersham Pharmacia Biotech (Piscataway, NJ, USA).

### Quantitative real-time PCR

Total RNA was isolated with the RNeasy Kit (Qiagen, Crawley, West Sussex, UK). One μg of RNA from each sample was reverse-transcribed with the QuantiTect^®^ Reverse Transcription (Qiagen). PCR reactions were performed in triplicate and fold changes were calculated with the formula: 2-(sample 1 ΔCt - sample 2 ΔCt), where ΔCt is the difference between the amplification fluorescent thresholds of the mRNA of interest and the mRNA of Glyceraldehyde 3-phosphate dehydrogenase (GAPDH) used as an internal reference.

### Elisa assay

Thyroid cells, MSCs and IMR90 (3 × 10^5^) were plated in six-well dishes, allowed to grow to 70% confluence, and then serum-deprived for 24 h. Culture media were centrifuged at 2000 rpm at 4°C to remove detached cells and debris. Attached cells were lysed, and total protein concentration was evaluated by a modified Bradford assay (Bio-Rad Laboratories), as described above. Shh levels in culture supernatants were measured using a quantitative immunoassay ELISA kit (RayBio Human ShhN ELISA Kit, Norcross, GA, USA) following the manufacturer's instructions. Triplicate samples were analyzed at 490 nm with an ELISA reader (model 550 microplate reader, Bio-Rad Laboratories). Shh levels, expressed in picograms per milliliter, were adjusted considering total protein levels of the grown cells.

### DNA constructs and reporter assay

pGL3-Gli-Luc was obtained by PCR-cloning eight copies of the Gli binding element into the pGL3 enhancer vector purchased from Promega. Luciferase activities present in cellular lysates were assayed using the Dual-Luciferase Reporter System (Promega, Madison WI, USA). In all cases, total amount of transfected plasmid DNA was normalized by adding *Renilla* luciferase reporter gene. Light emission was quantitated using a Monolight 2010 luminometer (Analytical Luminescence Laboratory). PSec-Shh construct has been previously described [38]. Data were represented as luciferase activity present in each sample and the values plotted were the average ± SEM of triplicate samples for typical experiments, which were repeated at least 3–5 times with nearly identical results.

### Cell proliferation assay

3 × 10^4^ cells were plated in 60-mm dishes. Cells were kept in RPMI 1640 or DMEM supplemented with 10% fetal calf serum. The day after plating, compounds or vehicle were added. Cells were counted in triplicate every 24 hours. To estimate IC50 value, cells were counted after 72 hours.

### Wound healing assay

A wound was induced on the confluent monolayer cells by scraping a gap using a micropipette tip. Photographs were taken at 10× magnification using phase-contrast microscopy immediately after wound incision and up to 24 hours later.

### Matrigel assay

*In vitro* invasiveness through Matrigel was assayed using transwell cell culture chambers. Briefly, confluent cell monolayers were harvested with trypsin/EDTA and centrifuged at 800 × g for 10 minutes. The cell suspension (2 × 10^5^ cells/well) was added to the upper chamber of transwells on pre-hydrated polycarbonate membrane filter of 8 mM pore size (Costar) coated with 35 μg Matrigel (Collaborative Research Inc.). Stromal cells (5 × 10^5^ cells/well) were plated on the bottom of wells. The lower chamber was filled with complete medium. Cell dishes were incubated at 37°C in 5% CO_2_ and 95% air for 48 hours. Non-migrating cells on the upper side of the filter were wiped off and migrating cells on the reverse side of the filter were stained with 0.1% crystal violet in 20% methanol for 15 minutes, counted and photographed.

### Spheroid-forming assay

50 cells per well were plated on ultra-low-attachment 96-well (Corning Incorporated, Painted Post, NY, USA) in low serum DMEM medium. Cells were maintained in a humidified atmosphere with 5% CO_2_ at 37°C. Spheroids were photographed after 7 and 14 days.

### Statistical analysis

Cell growth comparisons were done applying the Unpaired Student's *t*-test (Instat software, Graphpad Software Inc). All *P* values were two- sided, and differences were considered statistically significant at *P* < 0.01. IC50 values were calculated through a curve fitting analysis from last day values using PRISM software (Graphpad). P values were statistically significant at *P* < 0.01.

## SUPPLEMENTARY MATERIALS FIGURES


