# Supplementary material for: A dual mechanism of activation of the Sonic Hedgehog pathway in anaplastic thyroid cancer: crosstalk with RAS-BRAF-MEK pathway and ligand secretion by tumor stroma
Source: Oncotarget. 2017 Dec 17;9(4):4496–510. doi: 10.18632/oncotarget.23388 (PMC5796990; doi:10.18632/oncotarget.23388)
Supplement: Supplementary file 1 [file oncotarget-09-4496-s001.pdf]

A dual mechanism of activation of the sonic hedgehog pathway in anaplastic thyroid cancer: crosstalk with RAS-BRAF-MEK pathway and ligand secretion by tumor stroma

SUPPLEMENTARY MATERIALS

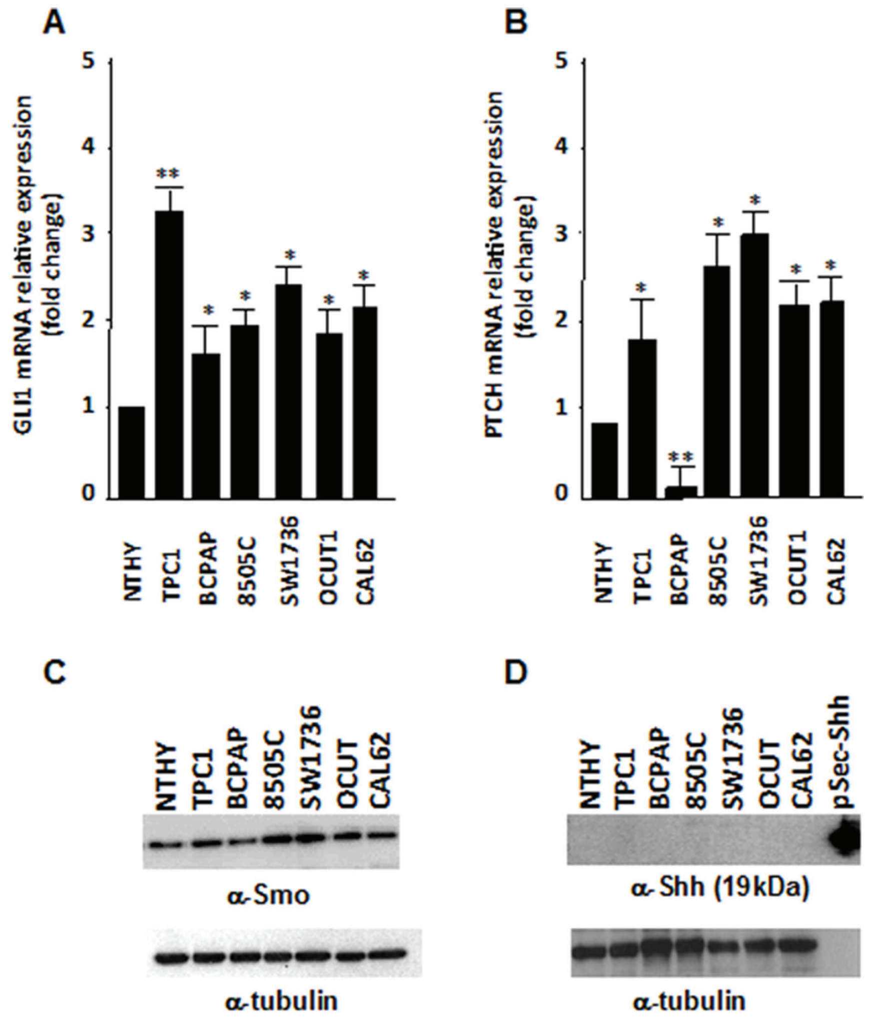

Supplementary Figure 1: Shh pathway components are expressed in thyroid cell lines. Q-RT-PCR for GLI1 (**A**) and PTCH (**B**) were performed in thyroid cancer cells and fold change with respect to NTHY was calculated. Error bars represent standard deviations of experimental triplicates. $^{**}p = \leq 0.01$; $^{*}p = \leq 0.05$. Smo (**C**) and Shh (**D**) expression was checked by Western blotting using specific antibodies. pSec Shh produced by HEK293T was used as positive control. Tubulin was used for normalization. The images are representative of three different experiments.

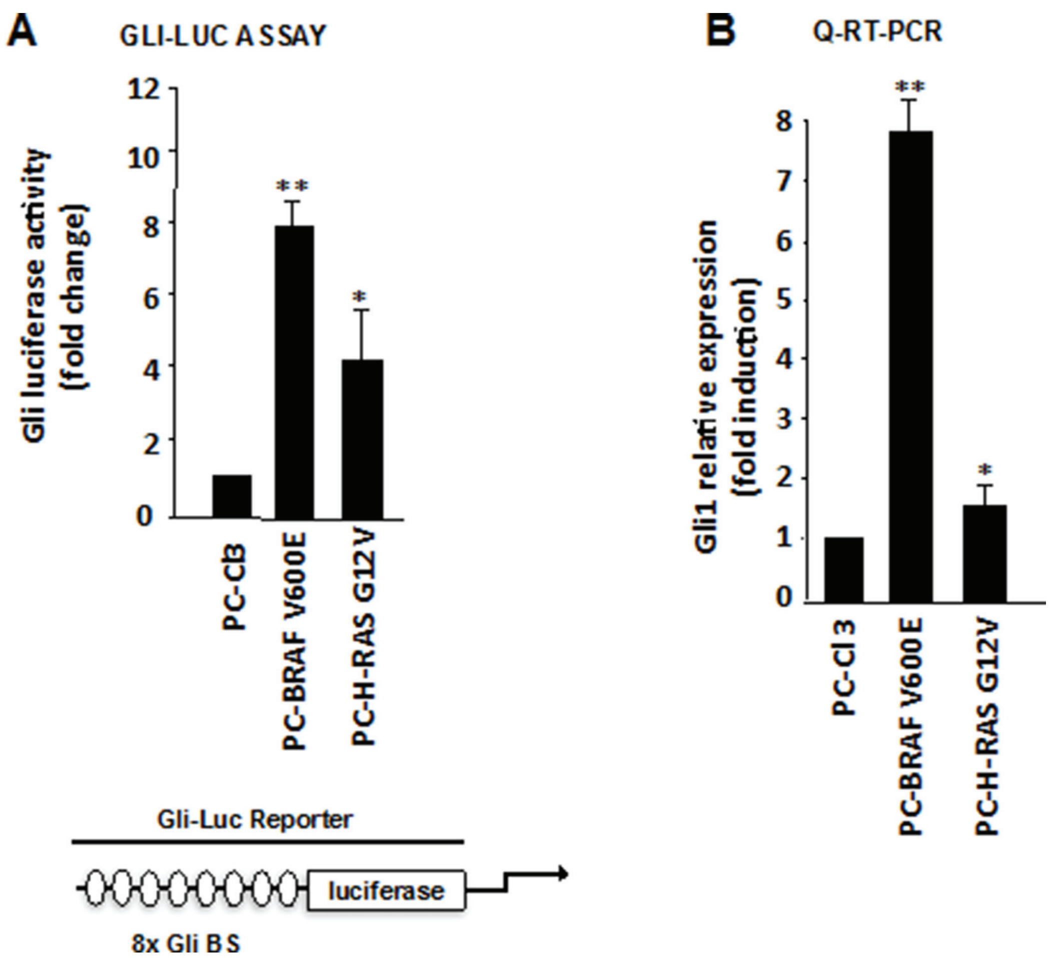

Supplementary Figure 2: Gli1 is up-regulated in PC cells stably transfected with BRAF V600E and HRAS G12V oncogenes. (**A**) Rat PC-Cl3 cells stably expressing activated BRAF (V600E) and RAS (G12V) oncogenic forms were transfected with Gli-Luc reporter (pGL3-Gli-Luc) and luciferase activity was measured 48 hours after transfection. Error bars represent standard deviations of experimental triplicates. (**B**) Gli1 mRNA expression levels were evaluated by Q-RT-PCR in PC-BRAF V600E and PC-HRAS G12V cells compared to PC control cells. Error bars represent standard deviations of experimental triplicates. $^{**}p = \leq 0.01$; $^{*}p = \leq 0.05$.

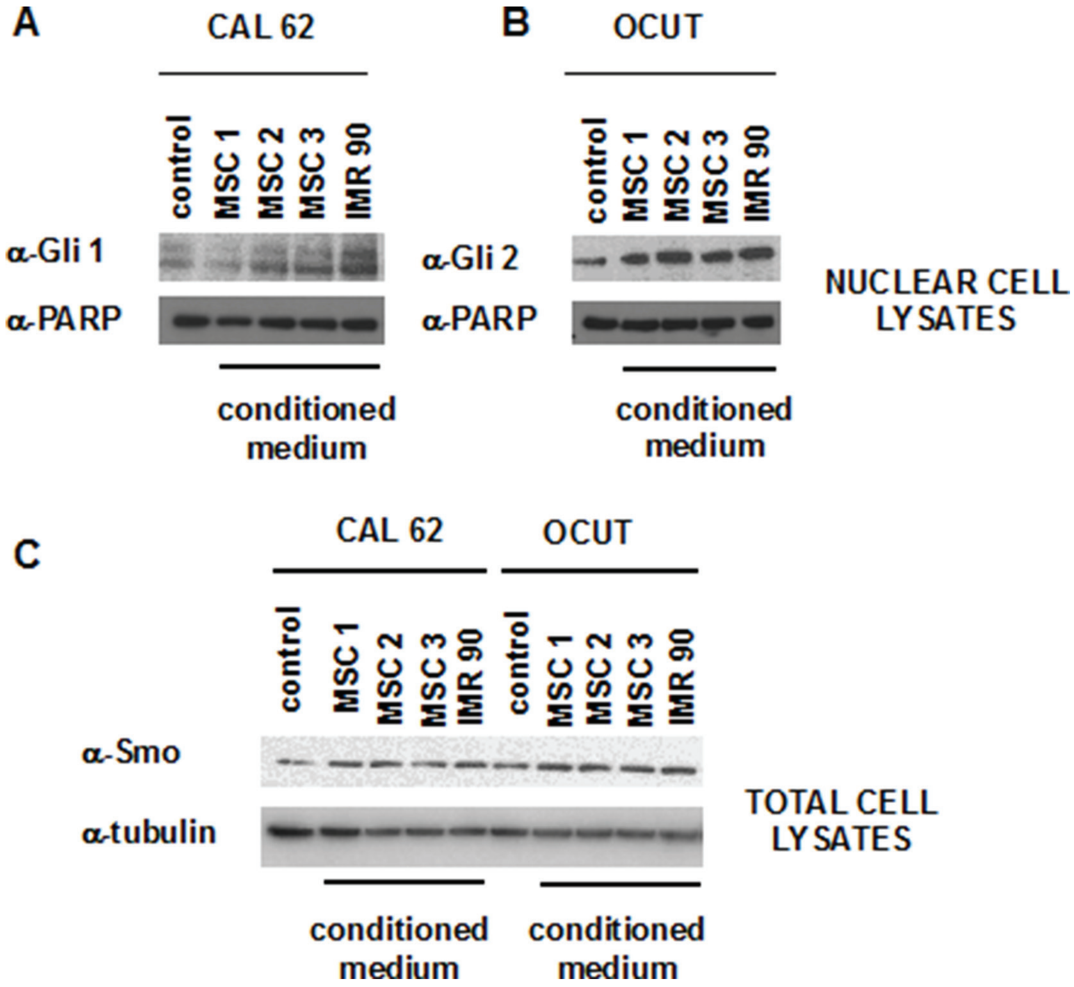

Supplementary Figure 3: Stromal cells stimulation induces up-regulation of Shh signaling components in anaplastic thyroid cancer cells. CAL62 and OCUT cells were stimulated with conditioned medium derived from IMR90 and MSC for 24 hours. After stimulation, cell fractionation was performed and nuclear cell lysates (30 μg) were analyzed with anti-Gli1 (**A**) and anti-Gli2 (**B**). Total Parp level was used for normalization. (**C**) Total cell lysates (50 μg) were analyzed with anti-Smo antibody and total tubulin level was used for normalization.
